# Camp Aruga: Building Professional Identity and Resilience in Filipino American Nurses

**DOI:** 10.1016/j.mnl.2025.102624

**Published:** 2026-02-12

**Authors:** Mary Dioise Ramos, Janette Moreno, Fe Rosario Antequino, Gloria Lamela Beriones, Manny Ramos, Marlon Garzo Saria, Lorraine S. Evangelista

**Affiliations:** Scholarship and Science, Louisiana State University Health Sciences Center –School of Nursing, New Orleans, Louisiana; Stanford Health Care, Stanford, California; Philippine Nurses Association of America, Inc, New Jersey; Primary Care Michael E. DeBakey VA Medical Center, Houston, Texas; Valencia College, Orlando, Florida; Philippine Nurses Association of America, Inc, New Jersey; University of California Irvine Sue & Bill Gross School of Nursing, Irvine, California

## Abstract

Camp Aruga Leadership Bootcamp, developed by the Philippine Nurses Association of America, incorporates Filipino cultural values into leadership training to boost professional identity, resilience, and retention among Filipino American nurses. This report provides an overview of Camp Aruga’s conceptual design, highlighting its main elements—culturally focused mentorship, ritual-based communal activities, nature-centered wellness, and practical governance and coaching workshops—along with the facilitators and challenges faced during its implementation. Results indicate the program successfully promotes self-care as a leadership practice, encourages interchapter collaboration, and strengthens cultural identity. Key challenges included logistical hurdles, the need for postevent follow-up structures, and limited resources for thorough evaluation. The findings provide an actionable and replicable model for culturally responsive leadership development.

Camp Aruga Leadership Bootcamp, an initiative of the Philippine Nurses Association of America (PNAA), embodies a leadership and wellness approach deeply inspired by Filipino cultural principles. The program intentionally integrates Filipino values, such as Aruga (care/nurture) and Bayanihan (communal solidarity), to address the persistent structural and psychosocial challenges faced by this work-force, including credentialing barriers, workplace marginalization, and acculturative stress.^1,2^ Research demonstrates that ethnic nursing organizations serve as crucial sources of connection, achievement, and buffers against racism and isolation, effectively supporting leadership development among minority nurses.^3^ Furthermore, mentorship programs designed explicitly for minority nursing populations have shown measurable benefits, with studies reporting 100% NCLEX pass rates among participants and evidence of early leadership uptake (25% of respondents obtaining leadership roles) following program participation.^4^

The program’s design is anchored in evidence demonstrating that culturally relevant supports, peer networks, and belonging rituals are crucial for enhancing professional identity, well-being, and workforce retention among immigrant and diaspora nurses.^5^ Camp Aruga strategically blends mentorship, rituals, nature-based wellness, and leadership training, offering a practical model to strengthen nurse resilience while honoring cultural identity. The curriculum intentionally combines formal learning with informal community-building, positioning rest, reflection, and mutual support as core leadership competencies.

The manuscript outlines the conceptualization, program design, implementation facilitators and barriers, and lessons learned from Camp Aruga, utilizing postevent participant comments and unstructured facilitator feedback to detail the program’s origins, core components, participant characteristics, and practitioner-reflection-based evaluation. Findings synthesize recurring themes—culturally anchored mentorship, ritualized communal practices, nature-based wellness, and practical leadership workshops—while identifying operational challenges, including remote venue logistics, variable chapter capacity for follow-up, and limited resources for longitudinal evaluation. The discussion translates these insights into actionable recommendations for nurse leaders, educators, and professional associations seeking to integrate cultural care and holistic well-being into leadership development.

## THEORETICAL FRAMEWORK AND UNDERPINNINGS

### Culturally Grounded Mentorship

Camp Aruga established its mentorship program as a reciprocal, culturally sensitive process designed to cultivate professional identity and leadership growth beyond the transfer of technical skills.^6^ Mentorship pairs and small groups, often supported by coaching frameworks like GROW and EMBRACE, set goals that integrate career advancement with culturally significant priorities.^7^ The deliberate use of rituals, storytelling, and community events celebrates migratory histories and transforms shared cultural values into actionable leadership strategies, offering a direct response to issues like limited leadership opportunities for Filipino American nurses.^6,8^

### Professional Identity and Well-being

The professional identity of Filipino American nurses is a convergence of immigrant experiences, bicultural socialization, and values like *Aruga* and *Pakikipagkapwa* (shared humanity).^9^ Programs like Camp Aruga create affirming environments where mutual mentorship and narrative exchange transform individual coping mechanisms—such as the resilience shown despite high nurse-to-patient ratios and acculturative stress—into collective professional assets. A report highlights the importance of culturally appropriate interventions, such as the PNAA’s “Kabalikat” initiative, that incorporate psychosocial and relational aspects of health, going beyond purely technical training.^10^

### Workforce Retention and Advancement

Filipino American nurses comprise an estimated 4.5% of the more than 3.8 million Registered Nurses in the U.S. Yet, they accounted for approximately 30% of the nurses who died from COVID-19 during the pandemic, underscoring their frontline role and risk exposure.^11^ Retaining this vital workforce requires effective strategies such as facilitating permanent residency, recognizing foreign qualifications, and offering culturally respectful, long-term mentorship.^12^ Formal programs that intentionally include leadership development, peer networking, and advocacy—like Camp Aruga—significantly increase the likelihood that Filipino American nurses will remain in the workforce and advance into positions of strategic influence, rather than leaving the profession due to burnout or marginalization.^7,8^

## DEVELOPMENT OF THE PNAA’S CAMP ARUGA PROGRAM

### Origins and Historical Context

The PNAA, founded in 1979, emerged from the efforts of Filipino nurses seeking mutual support, professional growth, and advocacy in response to ongoing issues, including under-recognition, unfair credentialing, limited leadership roles, and marginalization within major nursing organizations. Throughout its history, the PNAA has transformed into both a professional network and a custodian of the collective identity of Filipino American nurses, offering essential tools for acculturation, skill enhancement, and emotional well-being.

Camp Aruga, with ‘Aruga’ meaning ‘care’ or ‘nurture’ in Filipino, is rooted in this legacy. It is a culture-based leadership incubator that guides participants from their typical environment to restorative, nature-rich settings, encouraging reflection and community bonding. Iterative enhancements—guided by facilitator debriefs and participant feedback—have underscored the importance of scalability (regional cohorts), mentor training in coaching frameworks (such as GROW and EMBRACE), and pragmatic governance modules to connect individual development with organizational transformation.^13,14^ The program was conceived as a “transformative leadership development boot camp” designed to cultivate unity among chapters, improve leadership competencies, bolster resilience, and co-create a roadmap for health equity through a cultural lens.

### Evolution and Program Vision

Camp Aruga’s initial format, first piloted in 2023 and quickly expanded across PNAA’s 4 regional zones, integrated important lessons from mentorship and workforce retention research. It was intentionally designed to physically and symbolically separate participants from their usual environments, often in natural settings such as state or national parks, to encourage reflection, bonding, and peer learning. By 2025, Camp Aruga had been held multiple times in various U.S. regions, involving dozens of chapters in the Eastern, South Central, Western, and North Central PNAA areas, and drawing both early-career and seasoned nurses into its leadership and mentorship pipeline.

## PROGRAM COMPONENTS OF CAMP ARUGA

Camp Aruga weaves 5 interlocking components into each cohort: culturally anchored mentorship (formal dyads and small cohorts with coach-style training); ritualized communal practices (story circles, induction ceremonies, campfire reflections); nature-based wellness (guided hikes, sunrise reflections, movement sessions); practical leadership workshops (governance, advocacy, DEIB [diversity, equity, inclusion, belongingess], templates and scripts); and peer networking with continuity mechanisms (rotating-table project incubators, paired accountability, microcohorts, and follow-up check-ins). Table 1 illustrates the structure of the Camp Aruga program, which is organized around 5 interconnected pillars designed to develop culturally aware, resilient, and effective Filipino American nurse leaders.

## EVALUATING THE CAMP ARUGA PROGRAM

This program report used a descriptive, practice-focused qualitative approach to synthesize implementation lessons from Camp Aruga events held across multiple PNAA regions. Data sources encompassed unstructured participant comments from camp closeouts, facilitator debrief notes, open-ended feedback forms, and targeted narrative interviews with facilitators and selected alumni. These interviews were conducted to identify operational challenges and emphasize perceived benefits. The analytic emphasis prioritized recurring themes and practical relevance over prevalence counts.

An iterative thematic coding process was used. Two members of the program team independently reviewed and open-coded all textual materials. They then met to reconcile the codes and, through consensus, consolidate them into higher order themes. A third team member reviewed code definitions and exemplar quotations to improve consistency. Analysis centered on identifying facilitators, barriers, and actionable lessons tied to program components (mentorship, rituals, wellness, and leadership workshops) and on preserving divergent perspectives when present.

Ethical and contextual considerations: data were deidentified before analysis and shared only in aggregate or as anonymized illustrative comments. Findings are derived from purposive, program-based sources and are not intended to establish causal relationships. Limitations include the potential for possible selection bias toward more engaged participants and the lack of structured longitudinal survey data. Recommendations highlight practical, low-burden evaluation strategies to improve future evidence.

## RESULTS

Camp Aruga has attracted a broad spectrum of the Filipino American nursing community from across the country, bringing together nurses at different career stages, locations, and migration backgrounds. Attendees include early-career registered nurses and aspiring chapter leaders, midcareer supervisors, experienced nurse educators, and established clinical and executive leaders. While male participation is growing, it remains smaller than female participation, reflecting the wider workforce demographics. Geographic representation spans large urban PNAA chapters (for example, New York, Houston, and Los Angeles) as well as smaller regional chapters, creating opportunities for idea exchange between high-capacity hubs and community-based programs.

The participant group comprises both U.S.-born Filipino Americans and immigrant nurses, many of whom describe cycles of circular migration that link their professional paths with family and transnational connections. This variety of life experiences and migration backgrounds enhances peer learning by offering diverse viewpoints on credentialing, workplace integration, and community expectations.

Intentional inclusion strategies influence cohort composition and learning dynamics. Pairing new leaders with seasoned executives deliberately reduces hierarchical levels and encourages mutual learning. The program explicitly considers different generations by emphasizing communication styles and cross-generational mentorship. Recruitment spans clinical, academic, public health, long-term care, and advanced-practice settings, ensuring that disciplinary diversity informs governance, advocacy, and practice innovation. Together, these representational choices create a learning environment in which culturally grounded mentorship, practical governance skills, and wellness practices are tested and translated across a wide array of practice contexts.

## IMPLEMENTATION-FOCUSED THEMES (FACILITATORS AND BARRIERS)

Analysis revealed 6 primary implementation themes that directly influenced Camp Aruga’s ability to achieve its goals: culturally anchored mentorship, ritualized communal practices, nature-based wellness, practical leadership workshops, peer networking and continuity mechanisms, and operational barriers (remote-venue logistics and limited evaluation capacity). Each theme is summarized below, accompanied by supporting evidence and practical implications; deidentified exemplar quotations and linked implications appear in Table 2.

### Culturally Anchored Mentorship (Facilitator)

Formal mentor–mentee dyads and small-cohort coaching that explicitly incorporated cultural-reflection prompts converted participants’ migration and family narratives into actionable leadership goals. Facilitator notes and participant comments showed that pre-event mentor orientation and coach-style prompts increased the chances of follow-up actions on governance and advocacy after the camp. Participants described mutual learning, in which senior leaders shared systems knowledge and early-career nurses brought fresh perspectives, thereby boosting identity, confidence, and chapter results.

### Ritualized Communal Practices (Facilitator With Optionality Concerns)

Group rituals included a facilitator discussing optionality and featured activities like story circles, communal meals, initiation ceremonies, and symbolic handovers. These activities built shared meaning, mutual recognition, and cohort norms. These rituals enhance psychological safety and facilitate observable leadership behaviors. Analysis revealed differing comfort levels with ritual intensity, underscoring the need for precise framing, opt-out options, and facilitator emphasis on cultural safety.

### Nature-Based Wellness (Facilitator and Access-Related Barrier)

Guided hikes, sunrise reflections, movement sessions, and dedicated quiet-reflection periods produced restorative effects that increased openness and reflective learning; participants frequently described renewed permission to prioritize self-care as a leadership competency. At the same time, nature-based, remote sites introduced access, timing, and mobility challenges for some participants—logistical issues that occasionally undermined inclusivity and interrupted cohort cohesion.

### Practical Leadership Workshops (Facilitator With Scalability Needs)

Practical leadership workshops designed for facilitators with scalability needs. These short, case-based sessions on governance, advocacy, diversity, equity, inclusiveness, and belonging, along with coaching, included templates, scripts, and checklists that participants could use instantly in their chapters and workplaces. Many participants indicated they began implementing the workshop results locally shortly after the event. Facilitators observed that some chapters required additional technical support after the camp to advance their initiatives, underscoring the significance of scheduled follow-up help.

### Peer Networking and Continuity Mechanisms (Facilitator Contingent on Uptake)

Rotating-table project incubators, paired accountability, and microcohorts seeded collaborative projects, sustaining momentum through the adoption of continuity mechanisms. When sign-ups and scheduled check-ins were set before departure, microcohorts were more likely to persist and complete initiatives; when sign-ups were informal, projects frequently stalled.

### Operational Barriers: Remote-Venue Logistics and Evaluation Capacity

Remote, nature-based venue logistics repeatedly emerged as a practical barrier: transportation delays, mobility constraints, and communication gaps reduced participation in key opening rituals and occasionally necessitated program adjustments. Limited evaluation capacity—both in terms of staff time and limited dedicated funds—constrained the program’s ability to track outcomes beyond immediate reflections, limiting evidence for longer term funder or institutional support.

## IMPLICATIONS

Camp Aruga’s main strengths lie in deliberately combining culturally traditional practices—such as organized mentorship and ritual group activities—with practical skill-building elements. These include brief leadership workshops, templates, and scripts, supported by well-planned continuity strategies to sustain progress. These elements function synergistically: cultural practices create psychological safety and identity affirmation, increasing receptivity to skill-focused modules, and continuity mechanisms translate short-term gains into sustained chapter-level action. See Table 2 for deidentified exemplar quotes and the specific implications drawn from participant and facilitator feedback.

Key operational barriers clustered around logistics and capacity rather than program design. Remote, nature-based venues delivered restorative benefits but introduced consistent access, timing, and mobility challenges that reduced inclusivity and sometimes interrupted core rituals. Differential chapter capacity affected the uptake of postcamp continuity structures, causing promising projects to stall when follow-up pathways were informal. Limited evaluation resources restricted the program’s capacity to track long-term outcomes and show impact to funders and partners. Table 2 links these challenges with participant perspectives and specific implications.

## PRIORITY ACTIONS TO STRENGTHEN IMPLEMENTATION

To strengthen Camp Aruga while preserving its cultural integrity, program leaders should sequence a set of modest, low-burden changes that directly address the operational barriers identified in Table 2. Start by formalizing the mentor infrastructure: a brief, 60-90-minute orientation for mentors that includes cultural reflection prompts and a simple one-page goal contract, completed during the camp, will align expectations and increase postcamp follow-through. Additionally, provide a one-page follow-up checklist to help mentors maintain momentum with minimal administrative burden. At the same time, create a brief, optional menu of rituals with facilitator scripts and a cultural-safety briefing to ensure that rituals build trust without creating pressure; include clear opt-out options and brief adaptation guidance so local chapters can implement rituals safely and respectfully.

For wellness, focus on selecting restorative sites that meet basic accessibility standards or arranging transportation and assistive supports when necessary. Develop concise, repeatable wellness modules that can be utilized in more accessible settings when a site is challenging to navigate. Translate learning into action by bundling ready-to-use templates, scripts, and checklists in participant packets and scheduling 2 short postcamp technical-assistance sessions within 2-3 months to support immediate application and scaling. Make continuity automatic rather than optional by requiring participants to select a continuity pathway at registration and scheduling the first 3 check-ins before departure. Monitor progress with a simple alum project dashboard that minimizes reporting time.

Finally, adopt a pragmatic, phased evaluation plan: close out qualitative prompts, a brief 3-item follow-up at 3 months with a repeat at 6-12 months, and purposive alumni interviews to build a credible evidence base with minimal burden. Seek modest seed funding to support these evaluation tasks. Table 2 provides the deidentified exemplar quotations and specific implementation implications that informed each of these prioritized actions.

This program report compiles practical insights from different regional Camp Aruga iterations, reinforcing the idea that the observed patterns reflect consistent implementation dynamics rather than isolated site anomalies. Data triangulation involving participant closeout remarks, facilitator debriefs, open-ended feedback questionnaires, and purposive interviews enhanced analytic credibility and enabled the team to identify both prevailing patterns and divergent viewpoints. The analytical approach—using autonomous open coding, consensus reconciliation, and third-party auditing of code definitions and examples—enhanced consistency and reduced individual bias among coders. Focusing on tangible, low-effort implementation recommendations closely aligns with stakeholder requirements, making the findings readily actionable for program planners and chapter leaders.

## CONCLUSION

Camp Aruga demonstrates that culturally rooted mentorship, ritualized community activities, nature-based healing, and focused skill development can be merged into a practical model that enhances leadership skills among Filipino American nurses while maintaining cultural integrity. For Nurse Leader readers, the program offers a replicable template: combine brief mentor preparation and culturally tailored rituals with ready-to-use governance tools and built-in continuity pathways, reduce access and logistical barriers through site selection and transport support, and implement a phased, low-burden evaluation to measure impact. Implementing these prioritized, low-cost changes will help nurse leaders scale culturally responsive leadership development, strengthen chapter and organizational governance, and produce the evidence needed to secure institutional and funder support.

## Figures and Tables

**Table 1.** Camp Aruga Program: Core Components and Outcomes

Core Component	Key Activities	Primary Outcomes
*Culturally anchored mentorship*	Intentional pairing of early-career nurses/emerging leaders with experienced PNAA mentors, academic deans, and community leaders; formal and informal dyads/small groups; peer group mentoring; use of coaching frameworks (GROW, EMBRACE).	Increased confidence; Clearer professional identities; practical strategies for workplace impact; enhanced teamwork and lasting professional relationships.
*Ritualized communal practices*	Facilitated story circles; communal meals; induction ceremonies; symbolic item transfers (e.g., leadership handovers).	Identity affirmation; leadership enactment; Normalization of vulnerability; transformation of individual narratives into collective memory; Promotion of Filipino values (e.g., bayanihan, pakikipagkapwa); increased responsibility and accountability.
*Wellness activities and nature-based restoration*	Guided hikes; sunrise reflections; movement sessions; Designated quiet reflection periods; curriculum connecting rest to sustainable leadership practices.	Reduced cognitive load; improved psychological safety; opportunities for deeper peer mentoring; Conversion of emotional replenishment into continuous, low-effort routines; emphasis on self-care as a vital leadership skill.
*Leadership workshops and professional development*	Seminars on Filipino and American leadership principles; Engagement workshop; workshops on emotional intelligence and advocacy; DEIB (diversity, equity, inclusion, belonging) modules; Legal and governance training.	Strengthened cultural humility and integrity; enhanced self-awareness; practical skills to navigate complex organizational/regulatory settings; ability to promote cross-generational/cross-cultural understanding; competence in running nonprofit organizations.
*Peer networking and community building*	Integrated learning spaces (workshops to dining areas); Mixed format sessions (skill-building, small-group reflection, unstructured peer exchange, project-focused “hack” time); rotating table discussions; cross-chapter project incubators; post-camp follow-up.	Open sharing of learning; professional growth as relational; Widespread sharing of best practices; development of authentic relationships and strategic collaboration; increased likelihood of initiatives becoming actual changes.

PNAA, Philippine Nurses Association of America.

**Table 2.** Illustrative Quotes and Linked Implementation Implications

Theme	Exemplary Quotes	Implications for Improving Camp Aruga
*Culturally anchored mentorship*	“My mentor honored my story and showed me how to use it when advocating for change.” “Pairing with a chapter president helped me see a clear pathway to governance.” “Mentors asked about family expectations before career goals, which changed how I planned my next steps.”	Require a 60-90 min mentor orientation; include cultural-reflection prompts and a one-page goal contract; provide a brief mentor follow-up checklist.
*Ritualized communal practices*	“The evening circle made me feel seen and connected to others, leading change.” “Our induction ceremony made chapter responsibilities feel sacred and shared.” “Story circles helped me name struggles I’d hidden at work.”	Codify a short menu of optional rituals with facilitator guides, include cultural safety briefings, and provide adaptation notes for local contexts.
*Nature-based wellness and balance*	“The sunrise reflection gave me permission to rest and still be a leader.” “Walking and talking outside loosened the usual workplace defensiveness.” “After the hike, I committed to a weekly reflection practice at work.”	Standardize brief wellness modules and reflection prompts; select sites balancing restoration and accessibility; include contingency plans for mobility needs.
*Practical leadership workshops*	“I used the governance template from camp to propose a committee change the next month.” “The advocacy script helped me prepare for a difficult HR conversation.” “The bylaws checklist made our chapter election process smoother.”	Provide ready-to-use templates and scripts in participant packets; offer short post-camp technical assistance sessions; map workshops to concrete milestones.
*Peer networking and continuity*	“We left camp with a project plan, but without scheduled check-ins, it stalled at home.” “Our micro-cohort still meets monthly and has completed 2 initiatives.” “Paired accountability kept me engaged when work got busy.”	Build paired accountability and micro-cohort sign-ups into registration; schedule the first 3 postcamp check-ins before departure; create a simple tracking dashboard for alumni projects.
*Operational/logistics barriers*	“Late flights and shared rides made us miss key sessions.” “The trail was not safe for an older participant, and we had to change the plan.” “We don’t have the capacity to track alumni projects after 3 months.”	Publish travel itineraries at registration; designate a transport coordinator; vet sites for ADA access; budget modest travel stipends; adopt a phased low-burden evaluation plan.

## KEY POINTS

- Culturally anchored mentorship model: Structured dyads and small groups with mentor-style training that turn migration stories and family responsibilities into clear leadership goals.
- Ritualized community practices as essential elements: voluntary, formal rituals (story circles, induction ceremonies, symbolic handovers) that foster psychological safety and encourage visible leadership behaviors.
- Nature-based well-being included as a leadership skill: brief, repeatable restorative activities that transform self-care into an ethical leadership skill rather than a personal luxury.
